# Deciphering the Molecular Interactions of Tuberculosis and Colorectal Cancer: A Network and RNA-Seq Data Analysis Approach

**DOI:** 10.2174/0118715206431526251125092355

**Published:** 2026-04-15

**Authors:** Rongrong Yu, Ahmad Hasan, Muhammad Ibrahim, Wadi B. Alonazi, Bin Li

**Affiliations:** 1 College of Education, Zhejiang University of Technology, Hangzhou, 310032, China;; 2 Institute of Biotechnology, College of Agriculture and Biotechnology, Zhejiang University, Hangzhou, P.R. China;; 3 Health Administration Department, College of Business Administration, King Saud University, Riyadh, Kingdom of Saudi Arabia

**Keywords:** Tuberculosis, colorectal cancer, molecular interaction, transcriptomics, gene expression profiling, drug targets, biomarkers, immune response

## Abstract

**Introduction:**

A recent study revealed a correlation between TB and cancer, with individuals with a history of TB or current symptoms having a greater likelihood of developing colorectal cancer. This study aimed to explore transcriptomics data to identify new potential common therapeutic targets for CRC and tuberculosis.

**Methods:**

The GSE11199 dataset associated with TB and the GSE33113 dataset associated with CRC were retrieved from the Gene Expression Omnibus. The study identified commonly upregulated genes *via* R language, built a protein‒protein interaction network, and visualized it *via* Cytoscape, Cytohubba, and MCODE, revealing the role of miRNAs and TFs in regulating hub genes.

**Results:**

A total of 40 genes were found to be commonly upregulated, six of which were identified as hub genes, *i.e.* , *CXCL5, MMP3, MMP1, CXCL8, CXCL11* , and *SPP1* . In addition, 58 miRNAs and 28 TFs were found to be associated with the hub genes.

**Discussion:**

Our findings revealed that key genes associated with the tumor immune microenvironment such as *CXCL5* , an inflammatory chemokine; *CXCL8* , a neutrophil-attracting chemokine; *CXCL11* , which is chemotactic for activated T-cells; and *SPP1* , which promotes the recruitment of immune cells to the tumor microenvironment and is significantly linked with various miRNAs and transcription factors, could regulate the functions of these hub genes and contribute to the progression and pathology of CRC and TB.

**Conclusion:**

The identified genes hold strong potential to apprise the development of targeted therapeutic strategies and advance clinical applications for patients affected by both conditions.

## 1 INTRODUCTION

TB, often known as tuberculosis, is a transmissible bacterial infection triggered by *Mycobacterium tuberculosis* that mostly impacts the lungs but can also impact other parts of the body. TB has remained a significant global health issue in recent years, including 2024, with an estimated 1.9 billion individuals potentially infected, new cases around 10.6 million, and 1.6 million deaths. The disease can impact people worldwide, mostly in middle-income and low-income countries, where it is ranked as the seventh and eighth most common cause of death, respectively. These countries suffer the most from the TB epidemic, accounting for 80% of all cases globally [1, 2]. Mostly, TB occurs through small droplets in the air [3]. It is one of the worst illnesses and a threat to everyone worldwide [4, 5].

The incidence of TB has been gradually declining; however, the emergence of COVID-19 and other global political issues poses a threat to the smooth functioning of healthcare services and TB control programs, and the targets set by the global health community, such as the WHO, to eradicate TB remain unreached [6, 7]. TB is highly difficult, and clinical, radiological, bacteriological, or histological diagnosis cannot be guaranteed. The main usual treatment for TB is chemotherapy, but some patients are resistant to multiple drugs and exhibit extensive drug resistance. Advancements in genomics are revolutionizing the fight against TB by empowering researchers to better understand the *M. tuberculosis* genome, identify genetic variations, and develop personalized treatment [2, 8, 9].

Colorectal Cancer (CRC) is a prevalent global malignancy, with elevated incidence in developed countries such as North America, Australia, and Europe [10]. The lifetime risk is 1 in 23 for men and 1 in 25 for women, influenced by access to screening programs, lifestyle, and genetics [11]. Similarly, in most cancers, the early stages of CRC development are characterized by the expression of proteins involved in cellular replication [12]. DNA microsatellite instability, which subsequently results in DNA replication errors, is reported in up to 15% of CRC cases [13, 14]. Surgery is still the main mode of CRC treatment, but after surgery, the post-5-year mortality ratio is high. This is also because most patients with CRC tend, on average, to be diagnosed at a late stage. Therefore, the molecular mechanism implicated in carcinogenesis plays [14] a potential role in identifying CRC prognostic biomarkers and possible therapeutic anticancer drugs [15, 16]. A recent study across various populations presented at the European Congress of Clinical Microbiology and Infectious Diseases 2024 revealed that individuals with a history of TB are more likely to develop cancers, particularly lung, blood, gynecological, and colorectal cancers [17]. More recently, several genes and pathways related to TB-associated lung cancer, including the EGFR and PI3K–AKT signalling pathways, have been found to co-occur, highlighting the potential shared mechanisms that may contribute to both diseases [18, 19]. 

CRC and TB are both prevalent diseases; nevertheless, their simultaneous occurrence at the same site is rare, despite their potential to serve as differential diagnoses for one another. Carcinoma is typically more prevalent in the distal large intestine, whereas tuberculosis is frequently observed in the terminal ileum or ileocecal junction [20]. Intestinal TB is a form of extrapulmonary tuberculosis caused by *M. tuberculosis* that primarily affects the gastrointestinal tract. It usually results from ingestion of infected sputum in pulmonary TB, haematogenous spread from a primary focus, or direct infection through the intestinal mucosa [21, 22]. CRC is the predominant cancer of the gastrointestinal tract, with frequent occurrence in the sigmoid colon, rectum, cecum, and ascending colon. The prevalent forms of CRC include adenocarcinoma, mucinous adenocarcinoma, signet ring cell carcinoma, and small cell carcinoma. Certain disorders, such as ulcerative colitis, Crohn's disease, and schistosomiasis, increase the risk of cancer. Chronic inflammatory mucosal injury results in metaplasia, which subsequently progresses to dysplasia and ultimately neoplasia [23]. The invasion of dormant tuberculosis by cancer may result in the activation of tubercular lesions. Tumor antigens or peptides generated by tumors may disrupt the granuloma environment, facilitating the proliferation of *TB bacilli* [24]. Some studies suggest that any association between active tuberculosis and malignancy is due to the reactivation of infection in immunocompromised patients with cancer, rather than a causal relationship between infection and neoplasms. The correlation between intestinal tuberculosis and colon cancer is complex and contentious. Carcinoma-induced immune weakness may facilitate the invasion of *M. tuberculosis*, whereas prolonged mucosal injury might result in metaplasia, subsequently leading to dysplasia and carcinoma [17, 24].

The identification of common genetic factors linked with both TB and certain cancers could provide deeper insights into the underlying mechanisms of how immunosuppressive therapies might influence cancer development in individuals with a history of TB.In light of the above-mentioned hypotheses, the current study aimed to compare genes shared between tuberculosis and CRC using publicly available microarray datasets. The results revealed that CXCL5, MMP3, MMP1, CXCL8, CXCL11, and SPP1 are hub genes regulated by TFs and interacting with miRNAs. Overall, existing literature supports that transcriptomic data and hub gene functions reveal valuable opportunities for prognostic applications in both TB and CRC, as well as for the development of innovative anticancer therapies.

## 2 MATERIALS AND METHODS

All data processing steps and parameters used for analysis in this study, such as data acquisition, identification of DEGs, identification of protein‒protein networks to identify hub genes, identification of miRNAs, and identification of drug gene interactions, are presented in the graphical abstract.

### 2.1 Data Acquisition and Analysis

The datasets of two gene expression profiles for colorectal cancer and tuberculosis (GSE11199 and GSE33113) were retrieved from the GEO database [17, 24]. GSE11199 represents the tuberculosis with human macrophage gene expression profile dataset and contains 24 samples, including 12 control and 12 tuberculosis-associated samples. The dataset GSE11199 was selected because it specifically captures human macrophage gene expression profiles in response to M. tuberculosis infection, making it directly relevant for studying host–pathogen interactions. It contains a balanced sample distribution providing an equal comparison group for robust statistical analysis. Moreover, as a publicly available, peer-reviewed dataset in GEO, it supports reproducibility and facilitates integration with other omics data for comprehensive analysis. The GSE33113 dataset contains 96 samples, including 6 control samples and 90 CRC-associated samples. This dataset offers a robust representation of CRC variability across patients, strong statistical power to identify Differentially Expressed Genes (DEGs), and reduced risk of sample-specific bias. The use of human patient datasets ensures that the findings are more directly applicable to clinical scenarios, as they reflect the complexities and variations observed in actual patient samples.

Therefore, the GSE11199 dataset is associated with the expression profile of patients with tuberculosis infection, whereas the GSE33113 dataset is associated with the expression profile of patients with colorectal cancer. Three primary criteria were used for selection as outlined below: (1)"*Homo sapiens*" as a species selection control, (2) normal TB test, and TB-infected samples, and (3) type of test used (expression profiling by array). GPL 570 and GPL 570 Pearl were the chip platform's notes (ver. 5. 30). By employing GEOquery from the Bioconductor project and linear models for microarray data (limma) R packages, GEO2R was utilized to categorize DEGs for those samples that had two or more groups for comparison. Benjamini-Hochberg tests were carried out to control the false discovery rate. The selection criterion was a |log2 (FC)| > 1 for which the *p-value* was <0.05. The limma was employed to fit linear models to the expression data for each gene, comparing the expression between the defined groups. The Benjamini-Hochberg correction was employed to adjust p-values and regulate the false discovery rate, ensuring that the proportion of false positives among the identified DEGs remained at an acceptable level, while the absolute log2 fold change (|log2(FC)|) > 1 indicates at least a twofold change in expression between groups. A statistical significance threshold of a *p-*value < 0.05 was applied to ensure that only significantly DEGs could be considered.

### 2.2 Robustness Analysis via RRA

 The RRA method is a statistical approach that combines multiple ranking lists to identify elements that consistently rank at the top of these lists. By aggregating rankings from various sources, RRA aims to provide a more robust and reliable consensus ranking [25]. Several studies have reported positive outcomes following the analysis of the genetic lists derived from chip data. Consequently, the RRA approach was employed to identify DEGs across two datasets. By utilizing the R package RRA, genes consistently identified across both datasets were designated as robust DEGs. Subsequently, the FC rankings of the upregulated and downregulated genes in associated datasets were standardized. The genes with a p-value <0.05 and | log2 (FC) |>1 were chosen as the most significant DEGs. The genes believed to be the most significant DEGs are those with a *p-*value <0.05 and a | log2 (FC) |>1. Most therapeutic strategies aim to inhibit overexpressed drivers (*e.g.*, inflammatory mediators, oncogenes, proteases). Hence, prioritizing upregulated genes aligns with the translational goal of identifying targetable pathways, and downregulated genes were not included in the analysis, as they often reflect suppressed or secondary processes.

### 2.3 GO and KEGG Pathway Enrichment Analyses

Gene Ontology (GO) enrichment analysis is a critical method for understanding the functional implications of gene expression data [26]. GO enrichment analysis was performed on the identified DEGs to elucidate their potential biological significance, such as Molecular Function (MF), Cellular Component (CC), and Biological Process (BP) enrichment, *via* clusterprofiler and Enricher following the approach of Qian *et al.* [27]. Kyoto Encyclopedia of Genes and Genomes (KEGG) provides a systematic representation of molecular interaction networks, particularly through its KEGG PATHWAY database, which includes various pathway maps illustrating metabolic processes, drug development, genetic information processing, human diseases, environmental interactions, cellular processes, and organismal systems [28]. The KEGG pathways associated with the DEGs were analyzed *via* the clusterProfiler, which revealed a statistically significant difference (*p*<0.05) [29].

### 2.4 Network Analysis Through Protein–Protein Interactions

The identified robust DEGs were processed *via* the STRING online database to build and analyse the Protein-Protein Interaction (PPI) network of the shared genes in the two datasets [30]. Default parameter values, such as genes with a score ≥0.7, were used to build the network, as this threshold represents a moderate level of confidence in PPI. There are generally three kinds of confidence scores: scores < 0.4 indicate low confidence, scores between 0.4 and 0.7 indicate medium confidence, and scores > 0.7 indicate strong confidence. A high confidence score was opted to eliminate low probability and obtain reliable results. The PPI network was then visualized *via* Cytoscape software [31]. In the PPI network, notable nodes (proteins/genes) were identified based on a degree greater than 15. In the PPI network, key nodes (proteins/genes) were chosen on the basis of degree > 15. Cytoyscape's node degree indicates direct connections, with higher degrees indicating centrality. Setting the threshold at 15 or greater means identifying genes that interact with many other genes, suggesting that these hub genes could be key regulators or players in important biological processes. The MCODE plugin was utilized in the construction of the network's cluster modules, which are its primary components.

MCODE is a powerful tool in Cytoscape used to retrieve densely connected regions in large networks, with a node score cut-off = 0.2, a degree cut-off = 2, and K-Core = 2. A degree cut-off = 2 helps to eliminate weakly connected or isolated nodes, focusing on more connected and potentially influential nodes, thus refining the overall network structure, node score cutoff = 0.2 indicates that only nodes with a score above this threshold will be retained as it allows to filter out less significant nodes, retaining those with stronger potential biological relevance, and K-core = 2 ensures that each node in the sub-network has at least two connections, emphasizing more interconnected regions that may represent biologically relevant complexes [32]. The genes linked to the core network modules were primarily annotated using the KEGG signaling pathway and then analyzed in R. *P*-values < 0.05 were considered statistically significant. By applying a *p*-value cutoff of <0.05, we mean filtering out gene associations that are not statistically significant. This helps to focus on core network modules that are more likely to be truly associated with the biological process, increasing the reliability of our findings.

### 2.5 Screening of Hub Genes

As a central node, hub genes play a vital role in biological networks that regulate numerous other genes and biological pathways. Identifying these fundamental genes increases our knowledge of disease mechanisms, enables the identification of potential drug targets, and paves the way for targeted drug development and new therapeutic interventions [33]. The Cytoscape plugin cytoHubba is a powerful tool for identifying key nodes and subnetworks inside intricate biological networks. It provides an intuitive interface to elucidate indispensable network components involved in signal transduction, gene regulation, protein-protein interactions, and biological pathways [34]. This includes various topological analysis algorithms, including stress, intermediacy, eccentricity, centricity, entrainment, bottleneck, degree, Maximum Neighborhood Component (MNC), Maximum Click Centricity (MCC), Euclidean, Maximum Neighborhood Component Density (DMNC), percolated component (EPC), and six centricity metrics. These algorithms play a vital role in elucidating the centrality of genes within biological networks, ultimately leading to the identification of hub genes.

### 2.6 Drug-gene Interaction Network Analysis

The drug interaction network for hub genes for the exploration of drugs that target CRC and TB treatment was built *via* the Drug Bank network (https://go.drugbank.com), confirming the date and time in the event footer by using the Network Analyst web platform. The downloaded network file from Network Analyst was opened with Cytoscape. Network Analyst integrates data from DGIdb, allowing the mapping of potential drugs targeting each hub gene, the identification of therapeutic candidates, and the creation of a comprehensive view of the molecular landscape [35].

### 2.7 Regulatory Network of Transcription Factors for Hub Genes

Transcription Factors (TFs) are constantly the principal determinants in cell fate decision-making during homeostasis and the development of adult tissues. They are often associated with regulating the expression of hub genes, shedding light on the upstream regulatory mechanisms that might influence their role in a biological pathway or disease mode [36, 37]. JASPAR is a database that provides curated, high-quality collections of transcription factor binding profiles for multiple organisms [38]. The prediction by JASPAR was carried out based on position weight matrices representing the DNA-binding specificities of TFs. Candidate TFs were selected using the following key criteria: (i) restricted to *Homo sapiens* species to ensure biological relevance, (ii) TFs with high-quality, experimentally supported binding motifs were measured, and (iii) a relative profile score threshold of ≥80% was applied to minimize false positives while retaining biologically significant interactions.

Furthermore, TFs are proteins that can actively enable DNA replication and ensure that it is either initiated or inhibited prior to commencement. In other words, they determine whether the cell will initiate the process of replicating its DNA [39]. Conversely, miRNAs exert their regulatory effects at the post-transcriptional level, modulating gene expression after transcription initiation. They can regulate protein synthesis by preventing translation, usually by binding to miRNAs [40, 41]. The network of TF-miRNA regulators representing the hub genes was built by using RegNetwork [42]. For this study, the following selection criteria were applied: (i) only regulatory interactions specific to *Homo sapiens* were considered to ensure species relevance; (ii) TF–target relationships supported by experimental evidence or multiple independent prediction algorithms were prioritized; (iii) redundant or low-confidence associations were excluded to avoid noise; and (iv) the final TF set was filtered to include only those regulating the identified hub genes within the constructed gene network. The network was cut off at 1° and visualized *via* Cytoscape software.

### 2.8 Experimental Verification of Hub Genes via qRT-PCR

 Caco_2_ cells are an adherent epithelial cell line derived from colon tissue of a colorectal cancer patient and are widely used in biomedical research, particularly for studies of inflammatory processes, drug transport, and viral transfection. Moreover, considering the importance of Caco_2_ as a standard in vitro model for intestinal absorption and as a useful cell line in tuberculosis drug development, Caco_2_ was a considerable cell lines for CRC and tuberculosis [43]. Total RNA was extracted from both spore-treated and untreated Caco-2 cells using TRIzol reagent (TaKaRa Biotechnology, China) according to the manufacturer's guidelines. The concentration and quality of the extracted total RNA were determined using a NanoDrop 2000 spectrophotometer (Thermo Fisher Scientific, USA).

The extracted total RNA was subsequently used for cDNA library preparation. Six hub genes named *CXCL5, MMP3, MMP1, CXCL8, CXCL11*, and *SPP1* were identified as DEGs and further validated by RT-qPCR. Total RNA (2 μg) was used to synthesize cDNA following the manufacturer’s protocols. RT-qPCR was performed at 95°C for 10 seconds, followed by 40 cycles of 95°C for 5 seconds and 60°C for 30 seconds, using a SYBR-Green PCR Master Mix kit (TaKaRa Biotechnology, China) on a Bio-Rad Real-Time System (Bio-Rad, Hercules, CA, USA). The gene-specific primers used in this study are listed in Table **1**. The RT-qPCR analysis was performed independently in duplicate. The target genes were adjusted relative to the β-actin gene, serving as an internal control. The relative expression levels of the chosen genes were determined using the 2−ΔΔCt technique.

## 3 RESULTS

### 3.1 Data Preprocessing and Identification of Common DEGs

Among the DEGs, forty common upregulated genes were retrieved from the GSE11199 and GSE33113 datasets. These include CXCL5, INHBA, REG1A, CEMIP, MMP1, REG3A, CXCL8, FOXQ1, MMP3, CXCL8, CRNDE, FAP, SERPINB5, CLDN1, TREM1, CXCL3, ADAM12, GABBR1, DUXAP10, CTHRC1, CXCL11, SPP1, MMP12, CXCL6, SOD2, S100P, CXCL11, WISP1, DEFA5, CLDN1, ETV4, FCGR3B, SLC35D3, SLC7A11, EGFL6, PRRC2C, HCAR3, BGN, SLC6A14, and TACSTD2 (Figs. **1A**-**C**). Fig. (**1A**) represents Differentially Expressed Genes (DEGs) of tuberculosis *vs* control, Fig. (**1B**) represents DEGs of CRC *vs* control, and Fig. (**1C**) represents the shared and unique DEGs under both conditions.

### 3.2 GO and KEGG Enrichment Analyses of Shared DEGs

The results obtained from the GO analysis of DEGs showed extensive functional enrichment that could be divided into three main categories as described by Qian *et al.* [27]: biological process (Fig. **2A**), cellular component (Fig. **2B**), and molecular function (Fig. **2C**). For example, upregulated genes related to biological processes were primarily linked with the antimicrobial humoral response, extracellular matrix organization, extracellular structure organization, the humoral immune response, antimicrobial peptides, and the chemokine response. The cell components included the lateral plasma membrane, cell projection membrane, zymogen granules, and Golgi lumen. Most of the genes enriched in molecular functions were primarily associated with chemokine receptor binding, CXCR, cytokine activity, chemokine activity, signaling activator, receptor-ligand activity, and glycoprotein activity. The GO results demonstrate that the significantly enriched genes are heavily involved in immune responses, extracellular matrix regulation, and receptor-mediated signalling, with localization to membranes, extracellular structures, and secretory pathways. These findings highlight the dual importance of immune activation and tissue remodelling, which are central to both tuberculosis infection and CRC progression.

 KEGG pathway enrichment analysis revealed significant associations between the enriched pathways and interactions involving rheumatoid arthritis, cytokine-cytokine receptor interactions, the IL-17 signaling pathway, viral protein interactions with cytokines, cytokine receptors, the TNF signaling pathway, and pertussis (Fig. **2D**) (Table **S1**). Collectively, the enrichment results indicate that the upregulated genes are key regulators of signaling cascades, structural maintenance, and immune modulation, which may trigger the pathological connections between TB and CRC.

 The KEGG pathway analysis revealed that DEGs were strongly enriched in immune and inflammatory pathways, which are central to both tuberculosis infection and colorectal cancer progression. Additionally, pathways associated with cancer and chronic inflammatory diseases suggest that the shared molecular interactions between TB and CRC involve a complex interplay of immune activation, inflammation, and dysregulated cell signaling.

### 3.3 Module Analysis and Interpretation of the PPI Network

 This indicates moderate interconnectivity, with each node linked to roughly 3.5 others. The network density was close to zero, indicating a relatively sparse structure, while the clustering coefficient of 0.503 represented the degree of local interconnection among neighboring nodes. *CXCL8, CXCL5, CXCL6, CXCL3, and CXCL11* form the core chemokine cluster, which is highly interconnected and central to the network, whereas MMP1, MMP3, and MMP12 are well-connected and interact closely with chemokines and extracellular matrix-related genes. Overall, the PPI network revealed that the CXCL chemokines and MMPs are key molecular hubs in the interaction network, linking the immune response, inflammation, and extracellular matrix regulation processes that are central to both tuberculosis pathology and colorectal cancer progression.

 Additionally, the number of connections between nodes, analogous to a random model that displays the same features, is around 7, confirming the network's structured nature. A PPI enrichment having a *p*-value below 1.0 e-16 shows the obvious nonrandom characteristics of protein interactions within the network and indicates intricate biological or functional inter-connections among its constituents, as demonstrated in Fig. (**3**).

### 3.4 Uncovering the Potential Hub Genes Common to CRC and TB

PPI networks were employed to identify potential nodes using topological analyses such as betweenness, stress, closeness, radiality, eccentricity, bottleneck, MNC, MCC, EPC, degree, and DMNC, which were integrated in the CytoHubba plugin of Cytoscape. Consequently, a total of six hub genes were identified through network analysis, *i.e.*, *CXCL5, MMP3, MMP1, CXCL8, CXCL11,* and *SPP1*. Each of these genes was documented, along with its corresponding aliases and primary biological functions, as shown in Fig. (**4**).

### 3.5 Construction of the Gene–drug Interaction Network

Protein-drug interaction networks provide vital information that can guide the selection of suitable interventions for patients. A network of protein-drug interactions was collected from DrugBank, in which the Hub genes were predominantly associated with various drugs. In subnetwork 1, there are 7 nodes connected by 6 edges. Subnetwork 2 contains 23 nodes connected by 22 edges, forming a densely connected network. Among these nodes, one seed node stands out as a probable focal point or point of initiation for either network exploration or influence processes.

The protein-drug interaction network constructed from DrugBank and DGidb revealed that *MMP3, CXCL8, MMP1,* and *SSP1* are dominantly linked to various types of drugs (Fig. **5** and Table **S2**). Similarly, our findings, such as potential hub genes, may impact disease progression and drug resistance and help researchers understand hub genes in the context of complex biological networks.

### 3.6 Regulatory Network Demonstrating the Association between Transcription Factors and the Identified Hub Genes

 Subnetwork 1 consists of 35 nodes connected by 49 edges, demonstrating a denser structure than its node count would suggest. Among the 6 seeds found in this network, particular attention seems to be given to elements or entities that can be very powerful or significant. These seeds are the building blocks for establishing this network. The identification of the specific relationships between these seeds and other parts of the network, as well as patterns of behavior, may lead to the development of measures or strategies.

This pattern facilitates the identification of potential associations between various genes, such as *CXCL5*, which connects with 9 TFs; *CXCL8*, which connects with 2 TFs; *MMP1*, which connects with 4 TFs; *CXCL11*, which connects with 3 TFs; *MMP3*, which connects with 4 TFs; and *SPP1*, which connects with 6 TFs, as shown in Fig. (**6**).

### 3.7 TF-miRNA Regulatory Network Analysis

The TF-miRNA regulatory network represents the relationships among the hub genes, miRNAs, and TFs. Network Analyst Subnetwork 1 examined the TF-miRNA coregulatory network, which has 117 nodes and 143 edges and appears to be fairly connected. The ratio of edges to nodes indicates its dense structure, despite its modest size. With six seeds, the subnetwork is likely to have a core group of components that are central or prominent and could significantly impact network dynamics or information distribution.

The presence of seeds suggests this network may be poised to undergo a chain reaction of influence or information sharing. *CXCL10* was connected to 9 miRNAs, *MMP1* was connected to 5 miRNAs, *MMP3* was connected to 11 miRNAs, *CXCL5* was connected to 10 miRNAs, *CXCL8* was connected to 12 miRNAs, and *CXCL8* was connected to 11 miRNAs, as shown in Fig. (**7**). Therefore, the overall majority of the CX associated hub genes presented increased degrees of interaction and TF-miRNA regulatory network connections with family members.

Overall, the complex interactions between these genes, miRNAs, and TFs further emphasize the regulatory landscape governing gene expression in this network. miRNAs and TFs are well-known modulators of gene activity, and their involvement here suggests that these elements may serve as biomarkers for complex diseases. Specifically, *CXCL5,* with its high degree of interaction, has emerged as a promising gene regulatory target that is potentially pivotal for understanding disease progression and pathogenicity. This insight into the connectivity and interaction patterns of genes such as *CXCL5* may enhance our understanding of this disease at the molecular level, opening avenues for potential targeted interventions and biomarker development.

### 3.8 Validation of the Identified Hub Genes using qRT-PCR Analysis

To validate the results of the bioinformatics analysis, quantitative real-time PCR was performed using treated Caco-2 and untreated Caco-2 cell lines. Compared with those in spore-treated Caco-2 cells, the expression levels of *CXCL5, MMP3, MMP1, CXCL8, CXCL11*, and *SPP1* in spore-treated cells were significantly greater (*P* < 0.01) (Table **2** and Fig. **8**) and aligned with the *in silico* results. Moreover, real-time PCR data, such as gene expression peaks, can be requested at any time. The increased expression levels of the hub genes align with bioinformatics predictions, reinforcing the reliability of the computational analysis and highlighting their potential involvement in cancer progression and therapeutic resistance.

## 4 DISCUSSION

TB and carcinoma of the colon are common entities and are major concerns for all clinicians, as patients with these conditions often require immunosuppressive drugs, increasing their risk for opportunistic infections compared with the general population [44-46]. Consequently, identifying shared molecular signatures is crucial for providing effective treatments. The primary aim of our study was to identify common DEGs in TB and CRC, which may aid in predicting cases in which TB is complicated by CRC and in revealing potential mechanisms. The results of this study provide novel insights into the complex molecular interplay between tuberculosis and CRC by integrating RNA-Seq data analysis with network-based approaches. Notably, the presence of chemokine genes such as CXCL3, CXCL5, CXCL8, and CXCL11 highlights the critical role of chemokine-mediated immune cell recruitment and chronic inflammation in both TB infection and CRC development. Similarly, matrix metalloproteinases (MMP1, MMP3, and MMP12) and extracellular matrix-associated genes (CEMIP, CTHRC1, and BGN) point toward shared mechanisms of microenvironmental changes and tissue remodelling that may facilitate both bacterial persistence and tumor invasion. Moreover, genes such as S100P, SERPINB5, and CLDN1 are well documented in cancer biology, supporting their potential role as molecular drivers in the overlap between these two diseases [41, 47].

Network moderate-to-high-level clustering is measured by the coefficient of betweenness centrality, which indicates that nodes are more likely to be part of communities or clusters near their neighbors. Characteristically, the metrics provide valuable insights into the network's structure and connectivity [48]. Interestingly, in the PPI network, the average number of connections per node was approximately 7, a value considerably higher than that observed in a comparable random network model. This observation reinforces the nonrandom organization of the network and points toward functional clustering of proteins that may underlie key mechanisms common to both TB and CRC.

Among the various pathways associated with gene functions and drug interactions, key elements are strongly associated with CRC and TB, such as CXCR4, a chemokine receptor that binds the chemokine CXCL12 and triggers signalling pathways that promote processes such as cell migration and metastasis [49]. The cytokine superfamily known as tumor necrosis factor (TNF) stimulates signalling pathways that are necessary for cell division, survival, and death. The activation of transcription factors such as NF-kappaB and JNK can result from the recruitment of TRAF family proteins, which in turn promote immunological and inflammatory responses, cell survival, and differentiation [5, 50].

The functions of hub genes, such as CXCL5, CXCL8, CXCL11, MMP1, MMP3, and SPP1, were analysed. These genes are known to be involved in key functions common to both TB and CRC. For example, chemokines constitute a substantial family that mediates various biological processes, including chemotaxis, tumor formation, and angiogenesis, and are essential for initiating and coordinating the systemic and sequential recruitment and activation of cells in *M. tuberculosis*-infected lungs [51, 52]. Moreover, CXCL5, 8, or 11, chemokines secreted by pulmonary epithelial cells, play crucial roles in driving neutrophilic inflammation during TB infection [53]. Owing to these characteristics, CXCL subfamily genes may be useful as drug targets and biomarkers.

Matrix Metalloproteinases (MMPs) are a family of enzymes that cleave protein substrates *via* a common mechanism that activates a water molecule bound in the active site *via* a Zn^2+^ ion. Despite the structural similarity of the catalytic domain, MMPs exhibit significant variations in terms of their cellular and tissue localization, substrate specificity, membrane binding, and regulation. All members of the MMP family are associated with disease progression, particularly cancer metastasis, including CRC, chronic inflammation, resulting tissue damage, and neurological diseases [54. However, many cancer treatments targeting these enzymes have failed because of their toxicity and ineffectiveness [53, 54]. Nanodrug delivery systems that respond to the tumor microenvironment have demonstrated exceptional efficacy in tumor-targeted therapy, owing to their distinct targeting and drug-release properties [55]. The design of an enzyme-sensitive nanodelivery system that utilizes MMPs as targeted receptors could significantly enhance the efficacy of targeted medication delivery. Similarly, epigenetic modifications of histone acetylation regulate Mtb-dependent gene expression and MMP-1 and -3 enzyme secretion, potentially improving outcomes in TB drug resistance through host-directed therapy [56]. Mechanistically, MMPs, which can degrade lung collagen, have also been implicated in TB-associated lung tissue destruction. MMPs in tuberculosis orchestrate immune cell migration and granuloma formation, but when dysregulated, drive lung tissue destruction and cavitation [57]. Their activity lies at the interface of host defense and pathology, making them promising targets for diagnostics and host-directed therapies.

SPP1 stands for secreted phosphoprotein 1, more commonly known as Osteopontin (OPN). Secreted Phosphoprotein 1 (SPP1), also known as osteopontin, plays a crucial role in immune mechanisms and is important for developing many cancer types, including TB. High SPP1 expression is associated with shorter survivals. It is positively correlated with infiltrating CD8^+^ T cells, CD4^+^ T cells, neutrophils, macrophages, and dendritic cells. In cancer, particularly CRC, SPP1^+^ Tumour-Associated Macrophages (TAMs) promote tumour progression by supporting growth, angiogenesis, and immune evasion. These TAMs interact with T cells and stromal elements to maintain an immunosuppressive tumor microenvironment [58-60]. The strong correlation of SPP1 with the immune response suggests it could be a potential immunotherapeutic target in CRC and TB. During *M. tuberculosis* infection, SPP1 is secreted by macrophages, dendritic cells, and epithelial cells, where it acts as a chemoattractant, recruiting macrophages and monocytes to the infection site. It supports granuloma formation and stability by promoting cell adhesion and extracellular matrix interactions. In addition, SPP1 enhances Th1-type immune responses by increasing IFN-γ and IL-12 production, which strengthens macrophage activation and bacterial killing. While these functions are protective and essential for *M. tuberculosis*, excessive SPP1 expression may drive chronic inflammation, fibrosis, and tissue destruction, ultimately contributing to lung cavitation and disease progression [60, 61].

Further extensive analysis was carried out *via* a drug bank database and the literature. These results revealed that several drug–gene associations were related to both TB and CRC. Notably, pravastatin, sirolimus, and doxycycline showed dual benefits by enhancing autophagy or inhibiting MMP activity, thereby restricting *M. tuberculosis* while also suppressing CRC proliferation and angiogenesis. Agents such as interferon-β, ribavirin, tretinoin, and vitamin E have moderate effects through immune modulation and oxidative stress regulation [62, 63]. In contrast, corticosteroids and tacrolimus may be useful in TB management but pose risks of immunosuppression that could favor cancer progression. Overall, drugs that influence autophagy, MMP activity, and immune signaling have the strongest therapeutic potential across TB and CRC.

The complex process of gene expression is regulated by miRNAs and TFs and could be valuable biomarkers in complex diseases [64, 65]. Among the network interactions, CXCL5 had a high degree of interaction, with a value of 9, and could be a potential gene regulatory target for understanding the pathogenic nature of the disease [51, 66, 67]. Critical genetic variations in CRC exomes have also been identified and prioritized, with CTSB and CPNE1 emerging as the most significantly overexpressed and potentially pathogenic genes [68-70]. These findings highlight their potential as biomarkers for early detection and therapeutic targets, warranting further experimental validation.

Recently, a combination of gene coexpression networks and whole-exome sequencing was used to identify critical modules and recurrently mutated genes involved in the pathogenesis of TB-associated lung cancer [71, 72]. However, among the six hub genes identified, which are also common among TB and CRC patients, MMP3, MMP1, and SPP1 are those genes that have not been reported in previous studies but play a key role in the molecular mechanism of TB and CRC [73, 74]. Collectively, these findings highlight potential shared molecular pathways between TB and CRC, offering insights into their interconnected pathogenesis and possible therapeutic targets.

## CONCLUSION

To date, no genetic studies have compared the common genetic risk factors for tuberculosis and CRC. Through extensive analysis of gene interaction networks, we identified the intersecting molecular pathways and regulatory genes implicated in TB and CRC. The findings of our study provide exclusive insights into the genes common to these diseases. Consequently, this study revealed six hub genes, namely, CXCL5, MMP3, MMP1, CXCL8, CXCL11, and SPP1. CXCL5 showed a greater degree of regulatory network interaction and was validated *via* real-time PCR analysis. Overall, our study identified hub genes through network analyses, which led to the identification of potential biomarkers and drug targets. Moreover, integrating other omics data, such as metabolomics, proteomics, and epigenomics, can provide a more comprehensive understanding of the molecular mechanisms linking TB and CRC.

## STUDY LIMITATIONS

This study is the first to investigate shared genetic traits between TB and CRC; however, it is limited to transcriptomic data and partial validation. Additional experimental validation in larger cohorts and integration of other omics data (proteomics, metabolomics, epigenomics) are needed to fully elucidate the molecular mechanisms linking TB and CRC.

## Figures and Tables

**Fig. (1):**
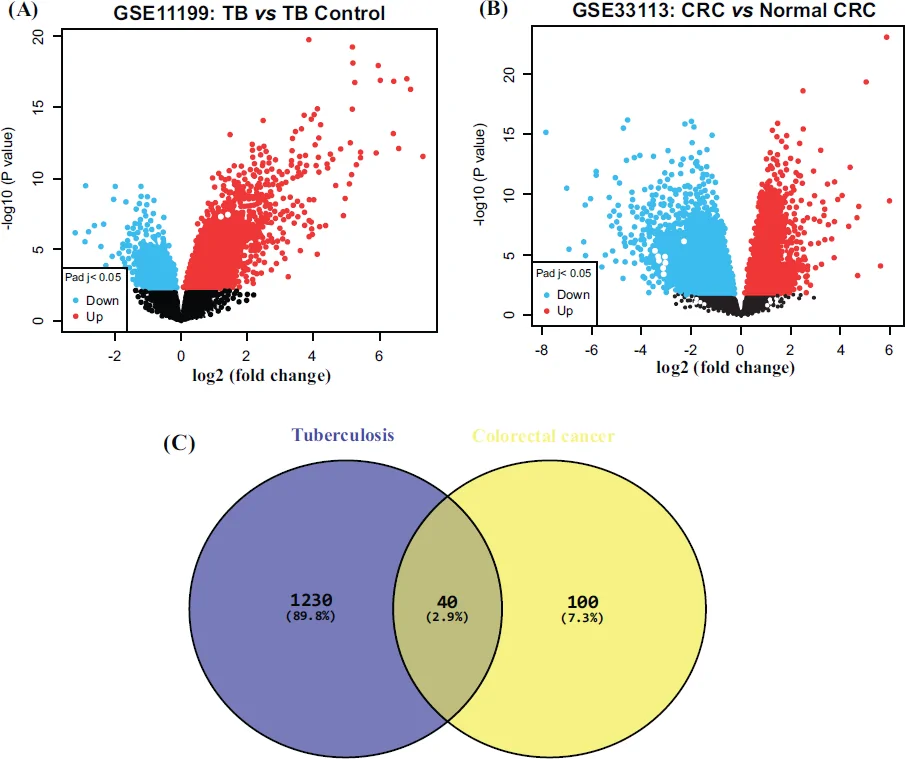
Identification and robust DEGs. Volcano plots describing the classification of DEGs in (**A**), GSE 11199; (**B**), GSE 33113; **(C)** Venn diagram of commonly identified upregulated DEGs among TB and CRC.

**Fig. (2):**
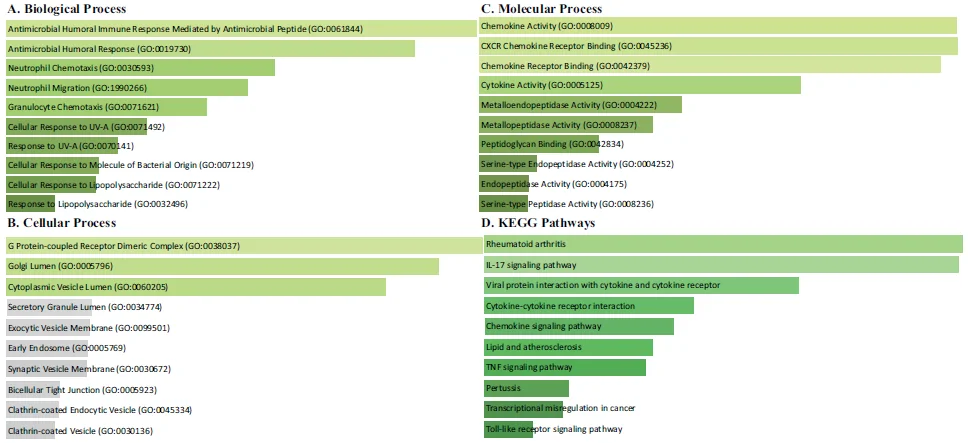
GO and KEGG enrichment results and analysis of commonly upregulated DEGs, (**A**) represents biological process, (**B**) represents cellular process, (**C**) represents molecular process, and (**D**) represents KEGG enrichment analysis.

**Fig. (3):**
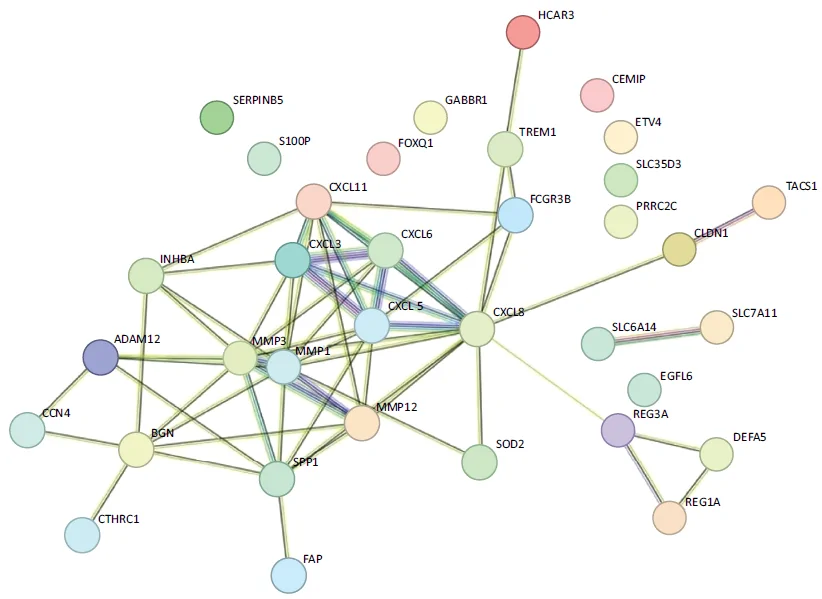
Protein-protein network interactions: Proteins are represented as nodes in the PPI network analysis graph; a thicker connection denotes a higher score, and a thicker line denotes greater protein interaction.

**Fig. (4):**
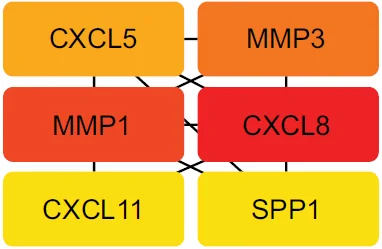
Network representation of the six hub genes identified through cytohubba analysis in cytoscape.

**Fig. (5):**
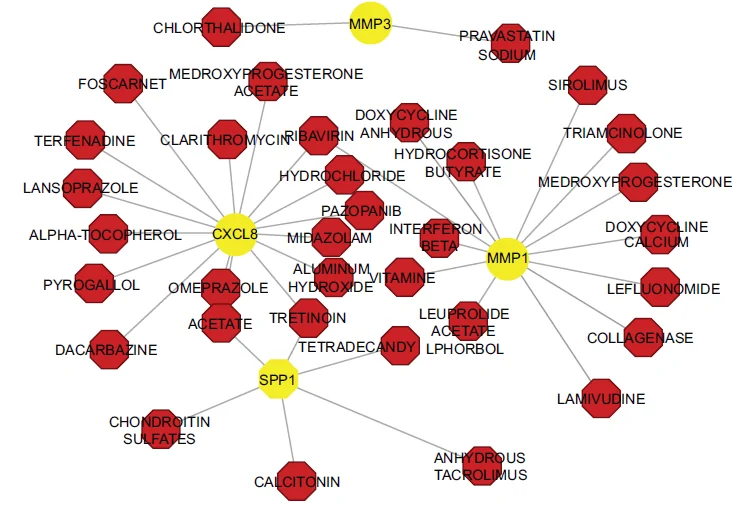
Protein drug interactions represent the core interaction with potential drug targets.

**Fig. (6):**
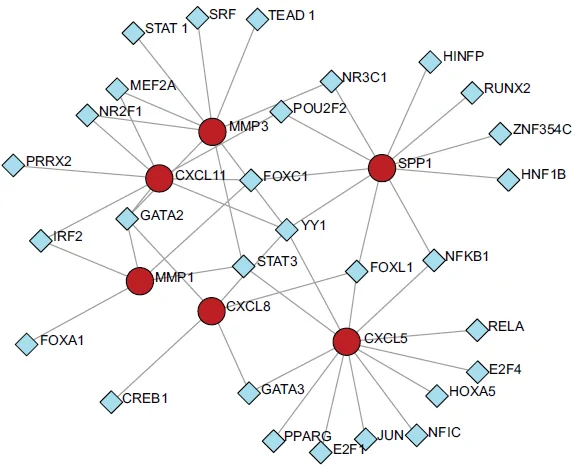
Network of transcription factor interactions regulating the shared hub genes. Nodes in red indicate hub genes, whereas other nodes represent TF genes.

**Fig. (7):**
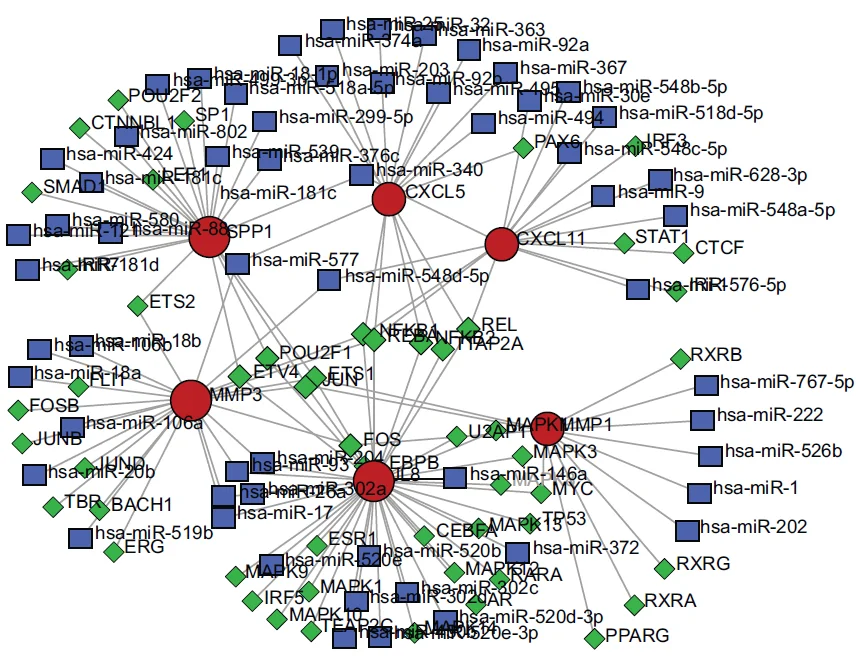
The TF-miRNA coregulatory network for common hub genes is indicated by the network's 144 nodes and 158 edges. This subnetwork also includes 8 seed nodes.

**Fig. (8):**
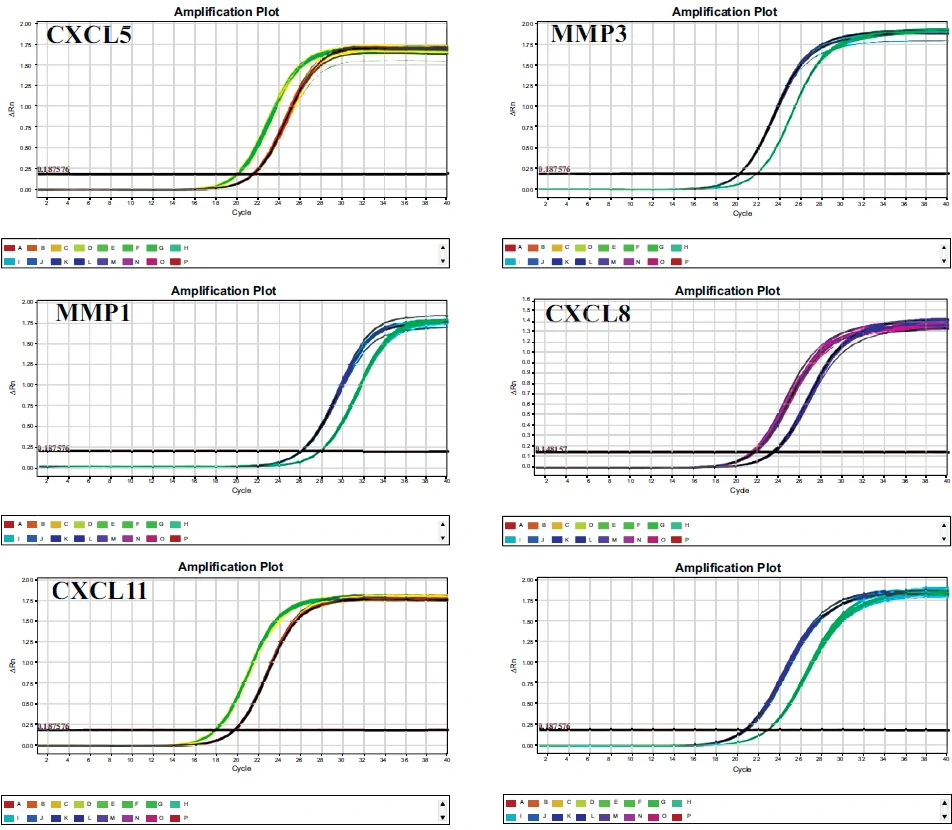
Amplification plot generated from quantitative real-time PCR, representing the quantification curves of six hub genes.

**Table 1:** Primer sequences of selected hub genes used for qRT-PCR validation.

**No.**	**GENE**	**SEQUENCE**
1	H-CXCL5-F	TGTTGCCATCAATGACCCCTT
	H-CXCL5-R	CTCCACGACGTACTCAGCG
2	H-MMP3-F	GGAAACCAGGAAGCCTAGCATC
	H-MMP3-R	GGATGATTCAGTGCCATTTTGCC
3	H-MMP1-F	AGTTGGAGGTCCAAAACGTG
	H-MMP1-R	ATTCTGAACCGGCTTGTGAC
4	H-CXCL8-F	GTGGAAGAGACTGCACAACAGC
	H-CXCL8-R	TCAGACGCTTGCTGATGGCTCT
5	H-CXCL11-F	GACCTGTGTCAGGCTTTCTCTG
	H-CXCL11-R	GGTATTTTGGTCTGACTGCTTGC
6	H-SPP1-F	GAGGTCCCAAAGATAAGCCAGG
	H-SPP1-R	CTGGCTTTATCCATCCACAGTCC
7	H-β-actin -F	GATGACGACCCCATGCAAAG
	H-β-actin -R	CGCACTTTCTCCGCAGTTTC

**Table 2:** Gene expression of hub genes via quantitative real-time PCR in Caco-2 cell lines and untreated Caco-2 cell lines.

**Hub genes**	**Expression Change Relative to that of the Control Conditions of the Cell Lines**	
	**Caco_2_**	**Cont.Caco_2_**
*CXCL5*	↑121.3	↑12.8 *
*MMP3*	↑12.3 *	↑16.1 *
*MMP1*	↑20.9 *	↑2.6 *
*CXCL8*	↑40.1 *	↑21.5
*CXCL11*	↑52.0 *	↑13.8 *
*SPP1*	↑66.0 *	↑15.1 *

**Note:** ↑: up-regulation; ↓: down-regulation; *: the change of gene expression is more than two-fold compared to the corresponding β-actin control.

## Data Availability

All data generated or analyzed during this study are included in this published article.

## LIST OF ABBREVIATIONS

BP: Biological Process
CC: Cellular Component
CRC: Colorectal Cancer
DEGs: Differentially Expressed Genes
GO: Gene Ontology
KEGG: Kyoto Encyclopedia of Genes and Genomes
MF: Molecular Function
