# Supplementary material for: Deciphering the Molecular Interactions of Tuberculosis and Colorectal Cancer: A Network and RNA-Seq Data Analysis Approach
Source: Anticancer Agents Med Chem. 2026 Apr 15;26(14):1349–62. doi: 10.2174/0118715206431526251125092355 (PMC13621376; doi:10.2174/0118715206431526251125092355)
Supplement: Supplementary file 1 [file ACAMC-26-14-1349_SD1.pdf]

SUPPLEMENTARY MATERIAL

Deciphering the Molecular Interactions of Tuberculosis and Colorectal Cancer: A Network and RNA-Seq Data Analysis Approach

Rongrong Yu[1], Ahmad Hasan[2], Muhammad Ibrahim[2], Wadi B. Alonazi[3] and Bin Li[2,*]

[1]College of Education, Zhejiang University of Technology, Hangzhou 310032, China; [2]Institute of Biotechnology, College of Agriculture and Biotechnology, Zhejiang University, Hangzhou, P.R. China; [3]Health Administration Department, College of Business Administration, King Saud University, Riyadh, Kingdom of Saudi Arabia

Table S1. The genes and their potential associated pathways.

ID	Description	p value	value	geneID	Count
hsa05323	Rheumatoid arthritis	8.2E-09	1.52E-07	CXCL5/MMP1/CXCL8/MMP3/CXCL3/CXCL6	6
hsa04657	IL-17 signaling pathway	8.75E-09	1.52E-07	CXCL5/MMP1/CXCL8/MMP3/CXCL3/CXCL6	6
hsa04061	Viral protein interaction with cytokine and cytokine receptor	6.56E-07	7.59E-06	CXCL5/CXCL8/CXCL3/CXCL11/CXCL6	5
hsa04060	Cytokine–cytokine receptor interaction	7.72E-06	6.7E-05	CXCL5/INHBA/CXCL8/CXCL3/CXCL11/CXCL6	6
hsa04062	Chemokine signaling pathway	1.64E-05	0.000114	CXCL5/CXCL8/CXCL3/CXCL11/CXCL6	5
hsa04668	TNF signaling pathway	4.25E-05	0.000246	CXCL5/MMP3/CXCL3/CXCL6	4
hsa05133	Pertussis	0.000319	0.001423	CXCL5/CXCL8/CXCL6	3
hsa05202	Transcriptional misregulation in cancer	0.000328	0.001423	CXCL8/MMP3/DEFA5/ETV4	4
hsa05417	Lipid and atherosclerosis	0.000494	0.001909	MMP1/CXCL8/MMP3/CXCL3	4
hsa04620	Toll-like receptor signaling pathway	0.000801	0.002781	CXCL8/CXCL11/SPP1	3
hsa05219	Bladder cancer	0.002444	0.007719	MMP1/CXCL8	2
hsa04621	NOD-like receptor signaling pathway	0.00424	0.012273	CXCL8/CXCL3/DEFA5	3
hsa05134	Legionellosis	0.004678	0.0125	CXCL8/CXCL3	2
hsa05120	Epithelial cell signaling in *Helicobacter pylori* infection	0.006983	0.017325	CXCL8/CXCL3	2
hsa05171	Coronavirus disease - COVID-19	0.007847	0.018171	MMP1/CXCL8/MMP3	3
hsa05146	Amoebiasis	0.014401	0.030533	CXCL8/CXCL3	2
hsa04064	NF-kappa B signaling pathway	0.014943	0.030533	CXCL8/CXCL3	2
hsa04936	Alcoholic liver disease	0.026838	0.051793	CXCL8/CXCL3	2
hsa05167	Kaposi sarcoma-associated herpesvirus infection	0.04753	0.084865	CXCL8/CXCL3	2
hsa05130	Pathogenic *Escherichia coli* infection	0.048862	0.084865	CXCL8/CLDN1	2
hsa04216	Ferroptosis	0.072295	0.119586	SLC7A11	1
hsa05144	Malaria	0.087497	0.138154	CXCL8	1
hsa04929	GnRH secretion	0.110684	0.167166	SPP1	1
hsa04622	RIG-I-like receptor signaling pathway	0.12207	0.1717	CXCL8	1
hsa04115	p53 signaling pathway	0.125298	0.1717	SERPINB5	1

hsa03320	PPAR signaling pathway	0.128515	0.1717	MMP1	1
hsa04512	ECM-receptor interaction	0.149158	0.185492	SPP1	1
hsa04350	TGF-beta signaling pathway	0.161633	0.185492	INHBA	1
hsa05150	*Staphylococcus aureus* infection	0.161633	0.185492	DEFA5	1
hsa05215	Prostate cancer	0.163181	0.185492	MMP3	1
hsa04933	AGE-RAGE signaling pathway in diabetic complications	0.167807	0.185492	CXCL8	1
hsa05142	Chagas disease	0.170878	0.185492	CXCL8	1
hsa04670	Leukocyte transendothelial migration	0.189082	0.199033	CLDN1	1
hsa04926	Relaxin signaling pathway	0.211312	0.207838	MMP1	1
hsa05135	Yersinia infection	0.222934	0.207838	CXCL8	1
hsa04371	Apelin signaling pathway	0.225815	0.207838	SPP1	1
hsa04550	Signaling pathways regulating pluripotency of stem cells	0.231546	0.207838	INHBA	1
hsa04072	Phospholipase D signaling pathway	0.238654	0.207838	CXCL8	1
hsa04932	Nonalcoholic fatty liver disease	0.248503	0.207838	CXCL8	1
hsa04218	Cellular senescence	0.2499	0.207838	CXCL8	1
hsa04514	Cell adhesion molecules	0.251295	0.207838	CLDN1	1
hsa05160	Hepatitis C	0.251295	0.207838	CLDN1	1
hsa05161	Hepatitis B	0.258232	0.208609	CXCL8	1
hsa04530	Tight junction	0.267844	0.20886	CLDN1	1
hsa05164	Influenza A	0.270569	0.20886	CXCL8	1
hsa04510	Focal adhesion	0.312899	0.236285	SPP1	1
hsa04024	cAMP signaling pathway	0.340658	0.246529	HCAR3	1
hsa05163	Human cytomegalovirus infection	0.340658	0.246529	CXCL8	1
hsa05131	Shigellosis	0.367368	0.256876	CXCL8	1
hsa05132	Salmonella infection	0.369746	0.256876	CXCL8	1
hsa05206	MicroRNAs in cancer	0.438376	0.298584	SERPINB5	1
hsa05165	Human papillomavirus infection	0.460342	0.307516	SPP1	1
hsa04151	PI3K-Akt signaling pathway	0.483478	0.316877	SPP1	1

Table S2. The drug-gene interaction and approval status.

Gene	Drug	Interaction Score	Relevance to Tuberculosis	Relevance to Colorectal Cancer
MMP3	Antihypertensive Agents	0.937637	No proper information	No proper information
	Pravastatin Sodium	0.277818	Enhances autophagy; restricts *M. tuberculosis*; reduces inflammation	Lowers CRC risk; inhibits proliferation, angiogenesis, and metastasis
	Chlorthalidone	0.8364562	Limited evidence; may indirectly influence immune response	No strong link; possible indirect effect via inflammation control
MMP1	Ribavirin	0.138178	Alters host immune responses, limited direct TB evidence	Explored for immune regulation; limited CRC-specific evidence
	Interferon Beta	1.312692	Potential to enhance immunity against TB	Inhibits tumor angiogenesis and proliferation
	Doxycycline Anhydrous	0.114147	Direct anti-MTB activity; reduces tissue destruction by inhibiting MMPs	Suppresses MMP1; inhibits angiogenesis and cancer stem-like cells
	Hydrocortisone Butyrate	0.125018	Adjunctive therapy in TB meningitis; risk of reactivation with prolonged use	May reduce inflammation but long-term immunosuppression increases cancer risk
	Leuprolide Acetate	0.525077	Minimal TB evidence	Alters hormone signaling; implicated in CRC progression
	Triamcinolone	0.228294	No proper information	No proper information

	Leflunomide	0.437564	Possible anti-TB role via immune modulation	Inhibits tumor growth and proliferation in CRC models
	Medroxyprogesterone	0.75011	No direct TB role	Hormonal modulation may influence CRC growth, limited evidence
	Sirolimus	0.092119	Enhances autophagy; restricts *M. tuberculosis*	Anti-tumor effect; inhibits CRC proliferation and angiogenesis
	Vitamin E	0.092119	Protects against oxidative damage in TB	Mixed evidence; protective at normal levels, controversial in high doses
	Lamivudine	0.437564	Minimal TB evidence	No established role in CRC
	Collagenase	0.75011	No direct TB use	Linked to ECM remodeling; overlaps with MMP biology in CRC
	Doxycycline Calcium	0.49098	No proper information	No proper information
CXCL8	Lansoprazole	0.013792	May alter gut microbiota and immune responses; limited TB evidence	Linked with microbiome changes and CRC risk modulation
	Ribavirin	0.117862	Alters immune response, limited TB role	Explored for immune regulation; limited CRC-specific evidence
	Omeprazole	0.076263		
	Dacarbazine	0.043216	No TB role	Used in oncology; inhibits DNA synthesis and proliferation
	Pazopanib	0.033529	No TB role	Inhibits angiogenesis and tumor growth; used in GI cancers
	Clarithromycin	0.0762638	Anti-MTB activity; modulates cytokines	Limited CRC evidence; possible microbiome-mediated effects
	Pyrogallol	0.043216	Possible antimicrobial activity	Induces apoptosis and ROS-mediated cancer cell death
	Tretinoin	0.014247	Enhances immune cell activation; limited TB evidence	Regulates cell growth and differentiation; anti-tumor effects in CRC
	Alpha-Tocopherol	0.6482	Protects against oxidative stress	Antioxidant; debated role in CRC
	Acetate	0.1296485	No proper information	No proper information
	Foscarnet	1.2964857	No TB relevance	Minimal CRC evidence
	Terfenadine	0.0720269	No TB relevance	Induces apoptosis in CRC models (off-target effect)
	Midazolam	0.0360134	No TB role	No direct CRC effect; supportive in cancer care
	Hydrochloride	0.259297	No proper information	No proper information
	Aluminum Hydroxide	0.346573	Enhances TB vaccine formulations	Used as adjuvant in experimental CRC immunotherapies
	Medroxyprogesterone Acetate	0.234765	No TB role	Hormonal modulation in CRC progression
SPP1	Tetradecanoylphorbol	0.250037	Induces inflammatory cytokines in TB	Promotes tumor progression via NF-κB/MAPK; experimental use
	Acetate	0.777891	No proper information	No proper information
	Anhydrous Tacrolimus	0.076934	Increases TB risk due to immune suppression	May suppress anti-tumor immunity
	Chondroitin Sulfates	0.466735	Limited TB role	Involved in tumor microenvironment remodeling
	Tretinoin	0.636457	Enhances immune activation in TB	Induces differentiation, suppresses proliferation in CRC
	Calcitonin	1.166837	Minimal TB evidence	Reported signaling effects, limited CRC evidence

Note: All the drugs interacted with hub genes are approved
